# Substance Abuse Among Adolescents in Urban Slums of Sambalpur

**DOI:** 10.4103/0970-0218.43236

**Published:** 2008-10

**Authors:** Lisa Sarangi, Himanshu P Acharya, Om P Panigrahi

**Affiliations:** Department of Community Medicine, V.S.S Medical College, Burla, Orissa, India

## Introduction

According to the World Health Organization (WHO),(1) substance abuse is “persistent or sporadic drug use inconsistent with or unrelated to acceptable medical practice”. The miasma of urban slums makes adolescents vulnerable to this multi-dimensional problem. Recently, substance abuse has been increasing among children and adolescents as sizeable proportions of adolescents in many states of India experiment with drugs quite early in life. This risk of drug abuse in adolescents can be conceptualized by the “Modified Social Stress Model”.(2) The model proposes that an increased risk for drug use stems from distress + the normalization of drug use + the effect of drugs and a decreased risk of drug abuse due to social attachments + coping strategies + resources for their development. This study has unveiled the current social problem with the objective of assessing the prevalence and type of substances abused in the urban slums of Sambalpur, Orissa and the various predisposing factors leading to their abuse.

## Materials and Methods

A cross-sectional, community-based study was conducted among adolescents of both sexes residing in the slums of Sambalpur, Orissa. The study was conducted from May 2004 to December 2005. A minimum sample size of 489 was calculated in accordance with the prevalence of substance abuse found in the pilot study conducted locally, which was 45% in adolescents of both sexes with a sampling error of 10% with a 95% confidence limit.

Out of 29 municipality wards in Sambalpur, one ward was selected by a simple random sampling technique. The slum area in that ward was identified. Each house was surveyed to register all the adolescent girls and boys among the slum population of that ward. The total number of adolescents identified was recorded. The same technique was continued in other wards until our calculated target sample size of 489 was reached i.e., a total of 502 adolescents were surveyed.

A house-to-house visit with the help of a pre-designed and pre-tested questionnaire with appropriate changes was conducted and the necessary information was collected. The statistical analysis was carried out using proportions, chi-square tests, odds ratio, and 95% confidence interval (Woolf's method).

## Results

Out of 502 adolescents who were surveyed (297 males and 205 females), 218 (43.4%) admitted to substance abuse with overall males abusing more 147 (49.5%) than females 71 (34.6%). The median age of substance abuse for males was 15.09 years old and 15.29 years old for females. The proportion of substance abuse was found to increase significantly (*P*<0.001) with age in both sexes, the highest being in the 16–19 year old group (55.2%). The most common substances used were Gutkha (91.7%), powdered tobacco (71.1%), tobacco toothpaste (Gudakhu) (63.8%), smoking (26.6%), and alcohol (14.7%). The substance abusers used multiple substances (3.34 substances abused per adolescent). Synthetic narcotics and LSD were not used by any of the abusers. Adolescents in joint families were consuming significantly higher amounts (47.3%) as compared with their counterparts in nuclear families (38.1%).

Thirty-four adolescents gave the history of their initiation to abusive substances before the age of 10. Gudakhu and pan were initiated more in the early adolescent age group. The mean age for the onset of use of Gudakhu is 14.65 years (± 0.63) and 14.98 years (±1.08) for pan, while smoking, alcohol, and cannabis are initiated more in the late adolescent period. The mean age for the onset of smoking, alcohol, and cannabis is 16 years (±1.5), 16 years (±1.5), and 16.75 years, respectively.

Substance abuse was found to be highest in broken families (51.2%) and the association was statistically significant (<0.02). A total of 186 (46.7%) adolescents who used substances have parents who both abuse substances and is closely followed by 43.5% of adolescents who have mothers who exclusively abuse substances. The influence of addicted peers was revealed in this study to be 48.3% with regard to initiation and continuation of substance abuse among adolescents, which is similar to the finding of Naskar, *et al.* (2004)(5) i.e., peer group influenced the initiation of substance abuse in 47.5% of substance abusers.

Adolescents found to be not using drugs had either father or neither parent abusing substances whereas adolescents with substance abuse had either both parents or only mother abusing substances [Table 1]. A significantly higher level of adolescents using drugs i.e., 139 (48.3%) had an addicted peer (*P*<0.02). The majority of substance abusers either never went to school (54.4%) or were school drop-outs (51.7%). The most common place for initiation of substance abuse was recreational avenues for males (49.7%) and home (36.6%) for females. A majority of the adolescents i.e., 150 (69.8%) purchase substances from their self earning.

**Table 1:** Association between risk factors and substance abuse among adolescents

Risk variable	Substance abuse among adolescents		
	
	With	Without	Odds ratio (95% CI)
Parents who abuse substances			
Both parents	186	212	3.2
No parent	5	18	(2.19–4.21)*
Child labor
Present	106	69	2.9
Absent	112	215	(2.52–3.28)
School attendance
Dropouts / illiterate	122	111	2.0
Regular	96	173	(1.65–2.35)*
Friends abuse status
Users	139	149	1.6
Non users	79	135	(1.24–1.96)*
Type of family
Joint	141	157	1.5
Nuclear	77	127	(1.14–1.86)*
Parental relationship
Broken family	43	41	1.2
Problem family	124	142	(0.71–1.69)*

* <0.05; CI = Confidence interval

Among the substance abusers, the most frequent compulsion was found to be peer pressure (52.8%). Among the females, substances were consumed most commonly for health reasons (42.3%). Among males, peer pressure remains the most common reason for substance abuse (63%).

A significantly higher proportion of substance abusers were associated with predisposing factors like joint family, parental abuse status, working status, and illiteracy/school-drop out. However, the odds ratio in support of a higher prevalence in broken families as compared with problem families was not statistically significant.

## Discussion

The present study reveals the prevalence of substance abuse to be 43.4%, which is lower than that reported in the slums of Bangalore(3) where the prevalence was 70.1%. This study also revealed that the most common substance being abused is the widely available Gutkha (91.7%). The smoking rate was higher among boys (35.4%) than girls (8.5%) although Sinha(4) had found prevalence of smoking to be 19.4% in school students of Bihar. A WHO study group on youth and drugs (1973) stated that most of the experimentation and initiation of dependence-producing drugs takes place during adolescence.

This study also finds that children belonging to broken families are the highest substance consumers (51.2%). This finding is similar to Benegal, *et al.* (1999)(3) who found 55% of slum children in Bangalore with substance abuse problems belonged to broken families. This study also reveals that a significantly higher number of adolescents resort to substance abuse when both parents are abusers (46.7%). The same impact was observed when only mothers consume substances (43.5%). A study in Bihar by Sinha(4) showed that 51.7% of school children abusing substances had a parent who smoked. The most common purpose of substance abuse in the present study was found to be peer pressure (52.8%) whereas Bansal, *et al.*(6) found the most common purpose of substance abuse to be curiosity or experimentation (34.3%) among child laborers from slums in Surat.

## Recommendation

This high prevalence especially due to high Gutkha consumption points out the necessity for a comprehensive strategy to curb the problem. Legislation on substance abuse has been vociferated loudly but still appears to be a mirage. Hence, a very pragmatic approach to containing the problem would be improved information education communication activities especially directed toward adolescents and their family members residing in urban slums.
